# Sanguinarine Suppresses Lung Adenocarcinoma *via* TGF-β1/Smad3 Path way: Insights from Network Analysis and Experimental Validation

**DOI:** 10.2174/0113816128404840251114063502

**Published:** 2026-01-09

**Authors:** Rong Yuan, Xiaona Li, Junqi Wang, Zhaojun Xu, Lan Song

**Affiliations:** 1 Medicine School, Hunan University of Chinese Medicine, 300 Xueshi Road, Hanpu Science and Teaching Park, Changsha, Hunan, 410208, China;; 2 Hunan Provincial Key Laboratory of Vascular Biology and Translational Medicine, 300 Xueshi Road, Hanpu Science and Teaching Park, Changsha, Hunan, 410208, China;; 3 Hebei Yuzhilin Biotechnology Co., Ltd., No. 168 Haidian Road, Shijiazhuang, Hebei, 050035, China;; 4 Cardiothoracic Surgery of the First Affiliated Hospital, Hunan University of Chinese Medicine, 97 Shaoshan Road, Changsha, Hunan, 410007, China

**Keywords:** Network pharmacology, molecular docking, bioinformatics, sanguinarine, lung adenocarcinoma, flow cytometry

## Abstract

**Introduction:**

This study elucidates molecular mechanisms underlying sanguinarine (SAN)-mediated inhibition of Lung Adenocarcinoma (LUAD) progression.

**Methods:**

Potential targets for SAN and LUAD were obtained from public databases. A Protein-Protein Interaction (PPI) network was constructed, and core targets were visualized using Cytoscape. Gene Ontology (GO) and Kyoto Encyclopedia of Genes and Genomes (KEGG) pathway enrichment analyses were performed with DAVID, while Reactome, WikiPathways, and MSigDB Hallmark analyses utilized Enrichr. Core targets expression and immune infiltration in LUAD were validated using The Cancer Genome Atlas (TCGA). Molecular docking assessed binding affinity between SAN and core targets, and *in vitro* experiments confirmed SAN's suppression of LUAD progression *via *the TGF-β1/Smad3 pathway.

**Results:**

Ten core targets of SAN in LUAD were identified. GO analysis revealed biological processes including proliferation, apoptosis, and signal transduction. Significantly enriched cancer-related pathways included PI3K-Akt, MAPK, Ras, and TGF-β signaling, the latter of which was significantly enriched across KEGG, Reactome, WikiPathways, and MSigDB Hallmark analyses. Molecular docking demonstrated a strong binding affinity between SAN and core targets. *In vitro* , SAN suppressed proliferation and autophagy in A549 cells while promoting apoptosis by inhibiting the TGF-β1/Smad3 signaling pathway.

**Discussion:**

The results demonstrate SAN's multi-target action against LUAD, notably through the inhibition of TGF-β1/Smad3, providing a mechanistic basis within oncogenic networks. Limitations include reliance on *in vitro* models and the preclinical focus. Future work requires *in vivo* validation and clinical translation.

**Conclusion:**

This study identifies key targets and pathways for SAN's inhibition of LUAD progression, validating its effect through the suppression of TGF-β1/Smad3 and providing experimental evidence for clinical application in LUAD therapy.

## 1 INTRODUCTION

Cancer remains the leading cause of mortality worldwide, with lung cancer exhibiting the highest incidence and fatality rates. According to World Health Organization (WHO) data, 2.5 million new lung cancer cases (12.4% of total cancer diagnoses) and 1.8 million lung cancer deaths (18.7% of cancer-related mortality) occurred worldwide in 2024 [1]. Non-Small Cell Lung Cancer (NSCLC) comprises approximately 85% of lung cancer cases, of which Lung Adenocarcinoma (LUAD) represents the predominant subtype. Despite current therapies significantly alleviating LUAD symptoms, treatment resistance and apoptotic evasion frequently lead to recurrence and poor prognosis [2]. Sanguinarine (SAN), a plant-derived alkaloid, has garnered significant interest due to its potent pharmacological properties, particularly its anti-tumor potential as depicted in Fig. (**1**).

This alkaloid is primarily sourced from plants in the Papaveraceae family, including *Chelidonium majus* L. and *Macleaya cordata* (Willd.). R. Br., Eomecon chionantha Hance, and the traditional North American plant *Sanguinaria canadensis* L [3-5]. SAN has shown potent effects in suppressing tumor cell proliferation, invasion, and metastasis, as well as in inducing apoptosis in cancer cells [6, 7]. The mechanisms underlying its action encompass various signaling pathways and molecular targets, such as Akt, Reactive Oxygen Species (ROS), and microRNAs (miRNA) [8-10]. Although SAN has been linked to LUAD pathogenesis [11], its therapeutic mechanisms remain incompletely characterized.

To address this gap, network pharmacology —an integrative approach combining bioinformatics and pharmacology—was employed to systematically identify drug-disease target associations and elucidate underlying mechanisms [12]. Here, we integrated network pharmacology and molecular docking to identify key targets of SAN in the treatment of LUAD. Subsequently, bioinformatics and *in vitro* assays are conducted to validate these targets and associated signaling pathways. This integrated strategy aims to establish a scientific foundation for pharmaceutical development and propose novel therapeutic approaches for LUAD.

## 2 MATERIALS AND METHODS

### 2.1 Targets Identification for SAN in LUAD Therapy

The experimental data for this study were partially sourced from online databases, with details of the databases, including their names and URLs, provided in Table **1**.

Using the Swiss Target Prediction database (probability > 0) [13] and PharmMapper [14], we identified 220 potential targets for SAN in LUAD treatment. LUAD-associated targets were retrieved from GeneCards (score > 20) [15], OMIM [16], and DisGeNET (score > 0.4) [17] with the query “lung adenocarcinoma”. All targets were converted to gene symbols using the UniProt database (https://www.uniprot.org/). Subsequently, we employed the online Venn analysis tool Venny 2.1 to map and visualize the overlap between SAN's active components and LUAD disease targets, identifying the common intersecting targets as potential therapeutic targets for SAN in LUAD treatment.

### 2.2 Construction and Analysis of the PPI Network for Potential Therapeutic Targets

The STRING database (https://string-db.org/) [18] generated a Protein-Protein Interaction (PPI) network for potential therapeutic targets. Intersecting targets from 2.1 were uploaded to STRING, and interaction data were exported in TSV format. Network topology was analyzed with Cytoscape 3.10.1 [19], with key targets identified by evaluating node degree, betweenness centrality, and closeness centrality. The top 10 targets were identified as core targets for SAN's therapeutic intervention in LUAD.

### 2.3 GO and KEGG Enrichment Analyses

Intersecting targets from Section 2.1 were analyzed using DAVID (https://david.ncifcrf.gov/home.jsp) for Gene Ontology (GO) terms and Kyoto Encyclopedia of Genes and Genomes (KEGG) pathway enrichment with “Homo sapiens” as the species. This analysis revealed potential pathways and biological processes associated with SAN's therapeutic effects on LUAD, and the results were visualized using a bioinformatics platform [20, 21].

### 2.4 Reactome, WikiPathways, and MSigDB Hallmark Analyses

Intersecting targets from Section 2.1 were analyzed using the Enrichr database (https://maayanlab.cloud/Enrichr/) for Reactome pathways, WikiPathways, and MSigDB Hallmark enrichment analyses. Results were also subjected to a comprehensive visualization analysis [22-24].

### 2.5 Molecular Docking Validation

The 3D structures of target proteins and the 2D structure of SAN were obtained from the RCSB Protein Data Bank (www.rcsb.org) and PubChem (pubchem.ncbi.nlm.nih.gov), respectively. Structures were refined with PyMOL and Chem3D 22.2.0. Molecular interactions were assessed using AutoDockTools 1.5.7 for docking, and the outcomes were optimally visualized with PyMOL [25].

### 2.6 Core Target Expression and Immune Infiltration Analysis

RNA-seq data from the TCGA-LUAD project, processed through STAR, were downloaded from the TCGA Data Portal (https://portal.gdc.cancer.gov), and TPM-formatted data were extracted. Core targets expression in LUAD patient tumors *versus* normal lung tissue was analyzed using R 4.2.1 with the stats and car packages. Additionally, the ssGSEA algorithm from the R package GSVA (version 1.46.0) [26] was employed to assess the relationship between core target expression and immune cell infiltration in LUAD, utilizing 24 immune cell markers derived from the Immunity journal [27].

### 2.7 In Vitro Experiments

#### 2.7.1 Materials

##### 2.7.1.1 Cell Lines

The human LUAD A549 cell line (purchased from Bei Na Chuang Lian Biotechnology, Beijing, China) was maintained in our laboratory through routine culturing.

##### 2.7.1.2 Main Experimental Drugs and Reagents

Ham's F-12K Medium complete culture medium (Procell, catalog number: WH1112004 XP), Fetal Bovine Serum (Gibco, catalog number: A3160802), SAN (Jinsui Biotechnology, catalog number: B21412), Cisplatin (Sigma, catalog number: P4394-25MG), and Annexin V-FITC/PI Apoptosis Detection Kit (Lianke Biotech, catalog number: 70-AP101-100) were utilized in the study.

##### 2.7.1.3 Main Equipment

Inverted Microscope (AE2000, Olympus Corporation, Japan), Automatic Microplate Reader (SN19092724, Bertin Instruments, USA), Multifunctional Fluorescence Imager (Odyssey CLX, LI-COR, USA), and Dual-Mode Fluorescence Cell Analyzer (Cellometer K2, Beijing Nuolaibio Biotechnology Co., Ltd.) were employed in the study.

#### 2.7.2 Methods

##### 2.7.2.1 Cell Culture

Human LUAD A549 cells were cultured in Ham’s F-12K medium supplemented with 10% FBS at 37°C under 5% CO_2_. Cells were subcultured at 1:3 upon reaching 80% confluency. Experiments utilized only healthy, log-phase cells.

##### 2.7.2.2 Monitoring Cell Proliferation with Real-Time Cellular Analysis (RTCA)

Culture medium was added to RTCA-specific plates, which were placed in the analyzer to establish a baseline measurement. Cells in the logarithmic growth phase were trypsinized to form a single-cell suspension and seeded into the plates. Monitoring commenced after parameter setup. SAN or cisplatin was added before the plateau phase, and monitoring continued for at least 72 hours thereafter.

##### 2.7.2.3 Cell Cycle Analysis

Cells were trypsinized to form a single-cell suspension at the appropriate concentration and plated in culture dishes. Once the cells had attached, they were treated according to experimental groups for 48 hours, trypsinized again, collected by centrifugation, and washed with PBS. After discarding the supernatant, the cells were fixed with 70% ice-cold ethanol and stained with a PI solution for flow cytometry.

##### 2.7.2.4 Autophagy Detection

Cells were trypsinized and plated in 6-well plates, and treated with drugs according to the experimental groups for 24 hours. Following a PBS rinse, monodansylcadaverine (MDC) staining solution was added, and the plates were incubated in the dark to stain. They were then washed with PBS and imaged immediately using a Cytation^TM^5.

##### 2.7.2.5 Annexin V-FITC/PI Double Staining for Apoptosis Detection

Cells were trypsinized and plated in 100 mm culture dishes, and treated with drugs according to experimental groups for 48 hours. Cells were trypsinized again, collected by centrifugation, washed with PBS, stained with Annexin V-FITC and PI, and analyzed using a fluorescence double-staining flow cytometer.

##### 2.7.2.6 Western Blotting

Cells were treated according to the experimental groups and washed twice with PBS. They were then lysed on ice with lysis buffer for 30 minutes, followed by centrifugation to collect the proteins. Quantified proteins were prepared for loading. Samples were electrophoresed on a 12% SDS-PAGE gel, transferred to PVDF membranes, blocked with 5% skim milk for 1 hour, incubated with primary antibody overnight, washed with TBST three times for 10 minutes each, incubated with secondary antibody for 2 hours at room temperature, and washed again with TBST three times for 10 minutes each. Finally, the membranes were scanned using the Odyssey-CLX system.

### 2.8 Statistical Analysis

 All data are expressed as mean ± SD (n=3 independent experiments). All data were statistically analyzed and processed using SPSS 21.0, while GraphPad Prism 8.0 software was utilized for data analysis and figure generation. The normality and homogeneity of variance assumptions for parametric tests were examined using the Shapiro-Wilk test and Brown-Forsythe test, respectively. For datasets satisfying both assumptions, statistical significance was determined by one-way ANOVA followed by Tukey's multiple comparisons test. In cases violating the normality assumption, the nonparametric Kruskal-Wallis test was employed, succeeded by Dunn's post-hoc test (or Mann-Whitney U test if applicable); *P*<0.05 indicated statistical significance.

## 3 RESULTS

### 3.1 Potential Targets for SAN Treatment of LUAD

We identified 220 potential targets for SAN in the treatment of LUAD using the Swiss Target Prediction and PharmMapper databases. Additionally, 1367 LUAD-associated targets were retrieved from GeneCards, OMIM, and DisGeNET (Table **S1**). Venn diagram delineated 54 intersecting targets indicative of SAN-LUAD interactions (Fig. **2**).

### 3.2 PPI Network Construction

The 54 intersecting targets were imported into STRING (https://string-db.org/) to construct a PPI network comprising 54 nodes and 500 edges. Subsequently, the TSV file was imported into Cytoscape 3.10.1 and filtered using the Centiscape 2.2 plugin, yielding 13 key targets based on topological parameters. The top 10 core targets ranked by degree value were MAPK1, IL2, EGFR, ALB, ANXA5, CASP3, HSP90AA1, ESR1, SRC, and JAK2 (Fig. **3**).

### 3.3 GO and KEGG Enrichment Analysis

GO analysis identified 180 significantly enriched terms (FDR ≤ 0.05), including 109 biological processes (BP), 20 Cellular Components (CC), and 51 Molecular Functions (MF), with the top 10 enriched terms visualized. KEGG analysis revealed 107 enriched signaling pathways (FDR ≤ 0.05), with visualization of the 10 most significant pathways depicted in Fig. (**4**). These analyses indicated that SAN treatment in LUAD primarily implicates biological processes such as "chromatin remodeling", "signal transduction", "negative regulation of apoptotic process", "positive regulation of cell population proliferation", and "positive regulation of cell migration". Key signaling pathways included "Pathways in cancer", "PI3K-Akt signaling pathway", and "MAPK signaling pathway".

### 3.4 Enrichment Analysis with Reactome, WikiPathways, and MSigDB Hallmark

Reactome, WikiPathways, and MSigDB Hallmark analyses identified 393, 305, and 21 significantly enriched terms, respectively (*P* < 0.05), with top 10 terms visualized in Fig. (**5**). These results associated SAN treatment in LUAD with pathways such as “Signal Transduction”, “Cancer Pathways”, “PI3K-Akt Signaling”, “MAPK Signaling Pathway”, “Ras Signaling Pathway”, “TGF-beta Signaling”, and “VEGFA-VEGFR2 Signaling”. Notably, TGF-beta Signaling was consistently enriched across KEGG, Reactome, WikiPathways, and MSigDB Hallmark pathways (Tables **S2-S3**), indicating a potential regulatory role of SAN in LUAD *via* TGF-β-related pathways.

### 3.5 Validation of Molecular Docking

Molecular docking validated core targets for SAN in LUAD treatment, demonstrating strong binding affinity (binding energies all less than -5 kcal/mol) as detailed in Table **2**, with structural interactions visualized in Fig. (**6**). These findings indicated spontaneous and stable binding between SAN and the core targets, supporting its therapeutic potential for LUAD.

### 3.6 Expression of Core Targets and Analysis of Tumor Immune Cell Infiltration

Bioinformatics analysis revealed significant expression differences for core targets, including MAPK1, IL2, ANXA5, CASP3, HSP90AA1, SRC, and JAK2, between normal and LUAD tissues (*P* < 0.05). Furthermore, these ten core targets exhibited significant correlations with the expression profiles of multiple immune cell types in LUAD. These analyses revealed a close association between core gene expression and immune cell infiltration, underscoring their crucial role in the immune microenvironment (Fig. **7**).

### 3.7 In Vitro Experimental Verification Results

#### 3.7.1 SAN Induces Cell Cycle Arrest via TGF-β1/Smad3 Pathway

SAN reduced TGF-β1 and Smad3 expression in A549 cells (Fig. **8A**). RTCA showed the TGF-β1/Smad pathway activator TGF-β1 (10 ng/mL) and inhibitor LY3200882 (15 μmol/L) modulated A549 cells' proliferation (Fig. **8B**). TGF-β1 increased PCNA expression in A549 cells *versus* controls. Moreover, combining TGF-β1 (10 ng/mL) with SAN (5 μmol/L) significantly elevated PCNA expression beyond SAN alone (Fig. **8C**). The working concentration of SAN (5 μmol/L) was selected based on our previous study (Figs. **S1** and **S2**). TGF-β1 (10 ng/mL) decreased the proportion of A549 cells in the Sub G1 phase, with the combination further reducing this proportion (Fig. **8D**). TGF-β1 (10 ng/mL) significantly enhanced CyclinD1, CyclinD3, and CDK4 expression in A549 cells. Combination therapy also increased these expressions compared to SAN alone (Fig. **8E**). Collectively, these findings suggest that SAN inhibits A549 cell proliferation by targeting the TGF-β1/Smad3 pathway, thereby arresting the cell cycle at the Sub G1 phase.

#### 3.7.2 SAN Suppresses Autophagy via TGF-β1/Smad3 Pathway

Fig. (**9A**) illustrates the fluorescence intensities: the blank group at 230.13 ± 3.48%, the TGF-β1 activator group at 253.33 ± 2.60%, the combination group with TGF-β1 + SAN at 215.57 ± 3.21%, the SAN group at 162.01 ± 5.29%, and the inhibitor LY3200882 group at 178.67 ± 5.21%. These results suggest that TGF-β1 significantly increased fluorescence intensity compared to controls; the TGF-β1 + SAN combination mitigated the inhibitory effect of SAN on A549 autophagy. Fig. (**9B**) shows TGF-β1decreased P62 and increased Beclin-1 and LC3II/I expression, while the combination reversed SAN-induced autophagy suppression (reduced P62; elevated Beclin-1/LC3II/I *vs.* SAN).

#### 3.7.3 SAN Promotes Apoptosis via TGF-β1/Smad3 Pathway

Fig. (**10A**) shows the apoptosis rates: the blank group at 1.12 ± 0.21%, the TGF-β1 activator group at 6.39 ± 0.58%, the TGF-β1 + SAN combination group at 21.07 ± 0.66%, the SAN group at 32.15 ± 0.81%, and the LY3200882 inhibitor group at 31.53±1.42%. These results suggest that TGF-β1 reduced A549 cell apoptosis compared to controls, while the TGF-β1+SAN combination attenuated apoptosis relative to SAN alone. Fig. (**10B**) indicated that TGF-β1 downregulated Bax expression and upregulated XIAP/ Survivin in A549 cells compared to controls; the combination group exhibited reduced Bax expression and elevated XIAP/ Survivin *versus* the SAN group, confirming that SAN induces apoptosis in A549 cells by inhibiting the TGF-β1/Smad3 pathway.

## 4 DISCUSSION

 SAN exhibits potent anti-cancer characteristics and may exert an effect on LUAD [28]. Network pharmacology, which integrates biology, pharmacology, and bioinformatics, provides a robust framework for elucidating the complex mechanisms of drugs. By integrating network pharmacology analysis with molecular docking validation, we identified ten core therapeutic targets of SAN in LUAD: MAPK1, IL-2, EGFR, ALB, ANXA5, CASP3, HSP90AA1, ESR1, SRC, and JAK2, all of which demonstrated spontaneous binding affinity with SAN. These findings corroborate the work of Saini *et al*. [29], who recognized SAN as a potent EGFR inhibitor with high bioavailability, favorable lead-like properties, low toxicity, and synthetic accessibility. Further bioinformatics analysis revealed the frequent dysregulation of these core targets in LUAD and their association with immune infiltration, suggesting that SAN may modulate the LUAD tumor microenvironment by regulating immune cell infiltration through these targets.

The GO enrichment analysis indicates that SAN influences LUAD through the regulation of proliferation, apoptosis, autophagy, and signal transduction. In 2020, Yu *et al*. [30] demonstrated that SAN suppresses macrophage activity by modulating LUAD-derived exosomes, thereby reducing LUAD cell metastasis and proliferation while enhancing apoptosis. Prabhu *et al*. [31] established that SAN induces LUAD cell apoptosis through reactive oxygen species (ROS) generation and JAK/STAT pathway inhibition, underscoring SAN’s therapeutic potential. Additionally, Cui *et al*. [32] identified SAN as a novel regulator of the Wnt/β-Catenin pathway that inhibits the polarization of M2 macrophages and angiogenesis, suggesting its potential in lung cancer immunotherapy and anti-angiogenic therapy.

Enrichment analyses (KEGG, Reactome, WikiPathways, and MSigDB Hallmark) indicate that SAN modulates multiple cancer-associated pathways, including the PI3K-Akt, MAPK, and Ras Signaling Pathways. Collective evidence from Zhang *et al*. [33], Lee *et al*. [34], Messeha *et al*. [8], and Qi *et al*. [35] demonstrates SAN’s PI3K-Akt-mediated anti-cancer effects. Xu *et al*. [36] confirmed SAN suppresses c-MET transcription and protein expression, subsequently inhibiting MAPK, PI3K/AKT, and STAT3 pathways to attenuate cell proliferation and induce apoptosis. Lee *et al*. [37] were the first to identify that SAN activates the Ras/ERK pathway through p27KIP1-mediated G1 phase cell cycle arrest, impacting cell growth. These studies collectively suggest that SAN may exert its anti-LUAD effects *via* these cancer-associated pathways.

Although PI3K-Akt, MAPK, and JAK/STAT pathways were enriched in our analyses, the consistent identification of TGF-β signaling across all databases, coupled with SAN's high binding affinity to TGF-β pathway-related proteins (Table **S4**, Fig. **S1**), motivated its experimental prioritization. We do not exclude roles for other pathways. SAN may act through parallel mechanisms, a hypothesis warranting future investigation. The TGF-β pathway is well recognized for its crucial role in tumor biology, where it is essential for inhibiting cell proliferation and inducing apoptosis to maintain normal cellular functions and prevent early tumorigenesis [38]. Studies indicate that genes associated with the TGF-β pathway play a modulatory role in tumor immune infiltration [39]. Smads, a key TGF-β effector [40], was investigated alongside TGF-β1. Western Blot analysis showed that SAN significantly reduced both the expression levels of TGF-β1 and Smad3 in A549 LUAD cells *versus* controls, indicating suppression of the TGF-β1/Smad3 pathway. However, it remains unclear whether SAN can modulate the TGF-β1/Smad3 signaling pathway to influence the initiation and progression of LUAD. To further investigate the functional impact of SAN, we treated A549 cells with TGF-β1 (activator) or LY3200882 (inhibitor) and assessed proliferation, cell cycle, autophagy, and apoptosis.

Consistent with our expectations, the combination treatment of TGF-β1 (10 ng/ml) and SAN (5 μmol/L) elevated TGF-β1 and Smad3 expression in A549 cells compared to SAN alone. Additionally, increased levels of PCNA, CyclinD1, CyclinD3, and CDK4, coupled with a reduction in Sub-G1 phase cells, indicated that SAN suppresses A549 cell proliferation and induces Sub-G1 arrest by inhibiting the TGF-β1/Smad3 pathway. MDC staining revealed significantly enhanced fluorescence intensity in A549 cells of the combination group compared to the SAN group, indicating increased autophagy. Furthermore, the expression of Beclin-1 and LC3II/I was elevated, while P62 expression was significantly reduced in the combination group, suggesting SAN inhibits autophagy in A549 cells by downregulating the TGF-β1/Smad3 pathway. Compared to the SAN group, the combination group exhibited increased expression of XIAP and Survivin, decreased Bax expression, and reduced apoptosis in A549 cells, suggesting SAN may promote apoptosis by suppressing the TGF-β1/Smad3 pathway to upregulate Bax and downregulate XIAP and Survivin in A549 cells.

Although our data demonstrate that pharmacological modulation of TGF-β1/Smad3 signaling alters the effects of SAN on A549 cells, the absence of genetic validation (*e.g*., Smad3 knockout/ overexpression) represents a limitation. Future studies employing siRNA, CRISPR/Cas9, or stable transfection models are needed to establish a definitive causal relationship between Smad3 expression and SAN efficacy. Meanwhile, it is important to note that our mechanistic validation was performed in A549 monocultures. While this model is widely adopted for initial mechanistic screening of LUAD therapeutics, it lacks the multicellular complexity and spatial heterogeneity of human tumors. Consequently, to strengthen translational relevance in future work, it‘s necessary to establish LUAD Patient-Derived Xenograft (PDX) models or murine xenografts with A549 cells to evaluate SAN efficacy in systemic contexts, monitoring tumor growth and TME remodeling.

## CONCLUSION

 In conclusion, network pharmacology and molecular docking analyses demonstrate the anti-LUAD effects of SAN, mediated through the regulation of LUAD cell proliferation, apoptosis, and multiple cancer-related signaling pathways. SAN likely interacts with core targets to exert these effects. Nevertheless, further studies are required to validate these findings. *In vitro* experiments demonstrate that SAN suppresses the proliferation of LUAD A549 cells by inhibiting the TGF-β1/Smad3 signaling pathway, inducing a sub-G1 phase cell cycle arrest, suppressing autophagy, and promoting apoptosis. This study establishes a mechanistic foundation for SAN in LUAD therapy and provides a scientific rationale for its clinical translation.

## AUTHORS’ CONTRIBUTIONS

The authors confirm their contributions to the paper as follows: RY was responsible for the study conception and design. JW handled the visualization. XL, ZX and LS drafted the manuscript. All authors reviewed the results and approved the final version of the manuscript.

## Figures and Tables

**Fig. (1):**
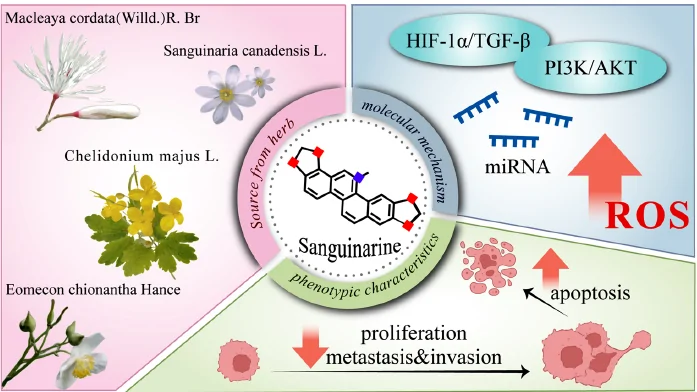
The source and pharmacological effects of SAN.

**Fig. (2):**
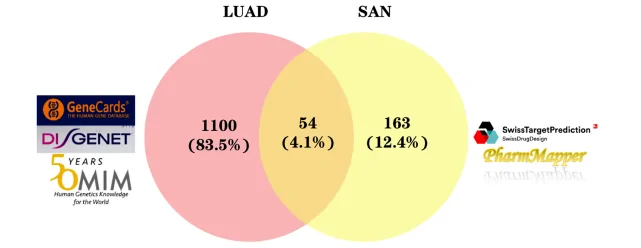
Venn Diagram of SAN interaction with LUAD targets.

**Fig. (3):**
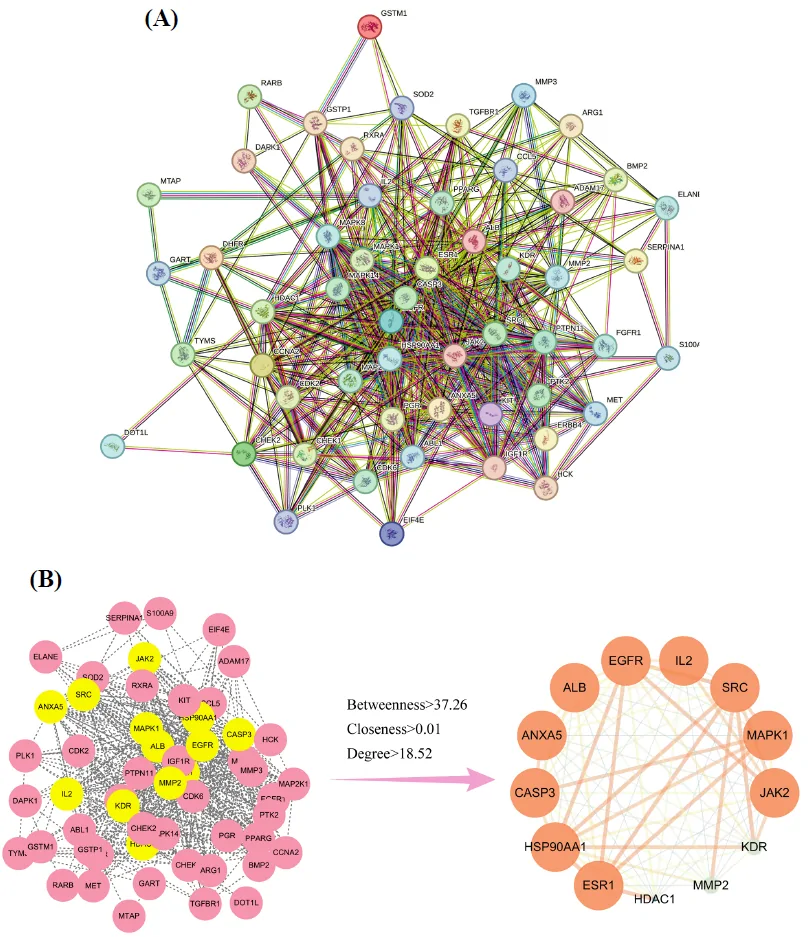
PPI Network (**A**) and Cytoscape visualisation (**B**) of targets for SAN treatment in LUAD.

**Fig. (4):**
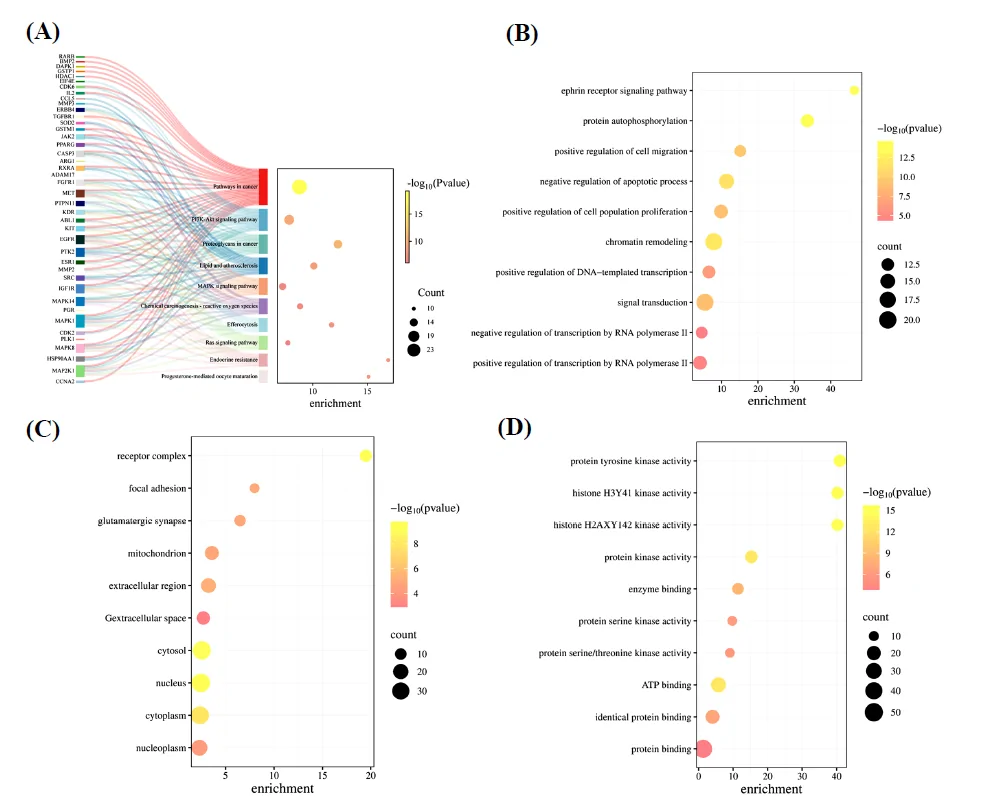
Enrichment analysis of SAN treatment in LUAD. (**A**) KEGG enrichment analysis; (**B**) GO-BP enrichment analysis; (**C**) GO-CC enrichment analysis; (**D**) GO-MF enrichment analysis.

**Fig. (5):**
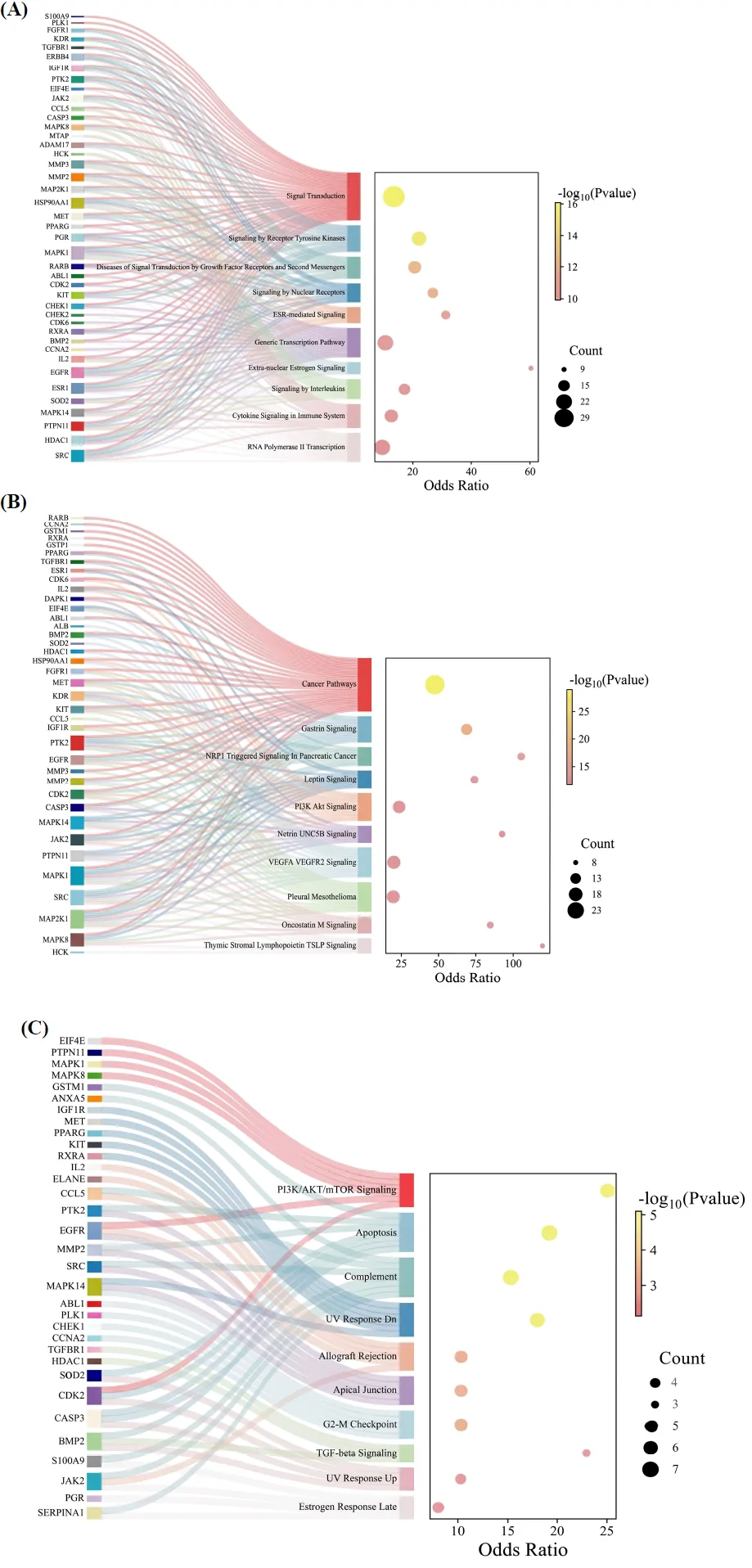
Enrichment analysis of SAN treatment for LUAD: Reactome pathways (**A**), WikiPathways (**B**), and MSigDB Hallmarks (**C**).

**Fig. (6):**
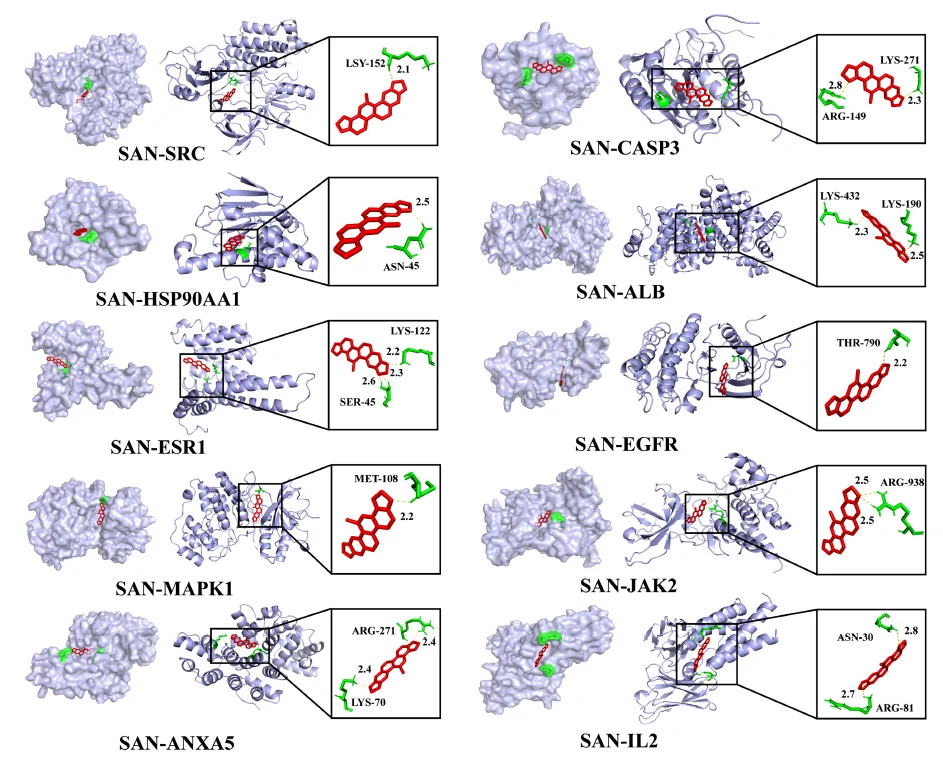
Molecular docking visualization of SAN with core targets.

**Fig. (7):**
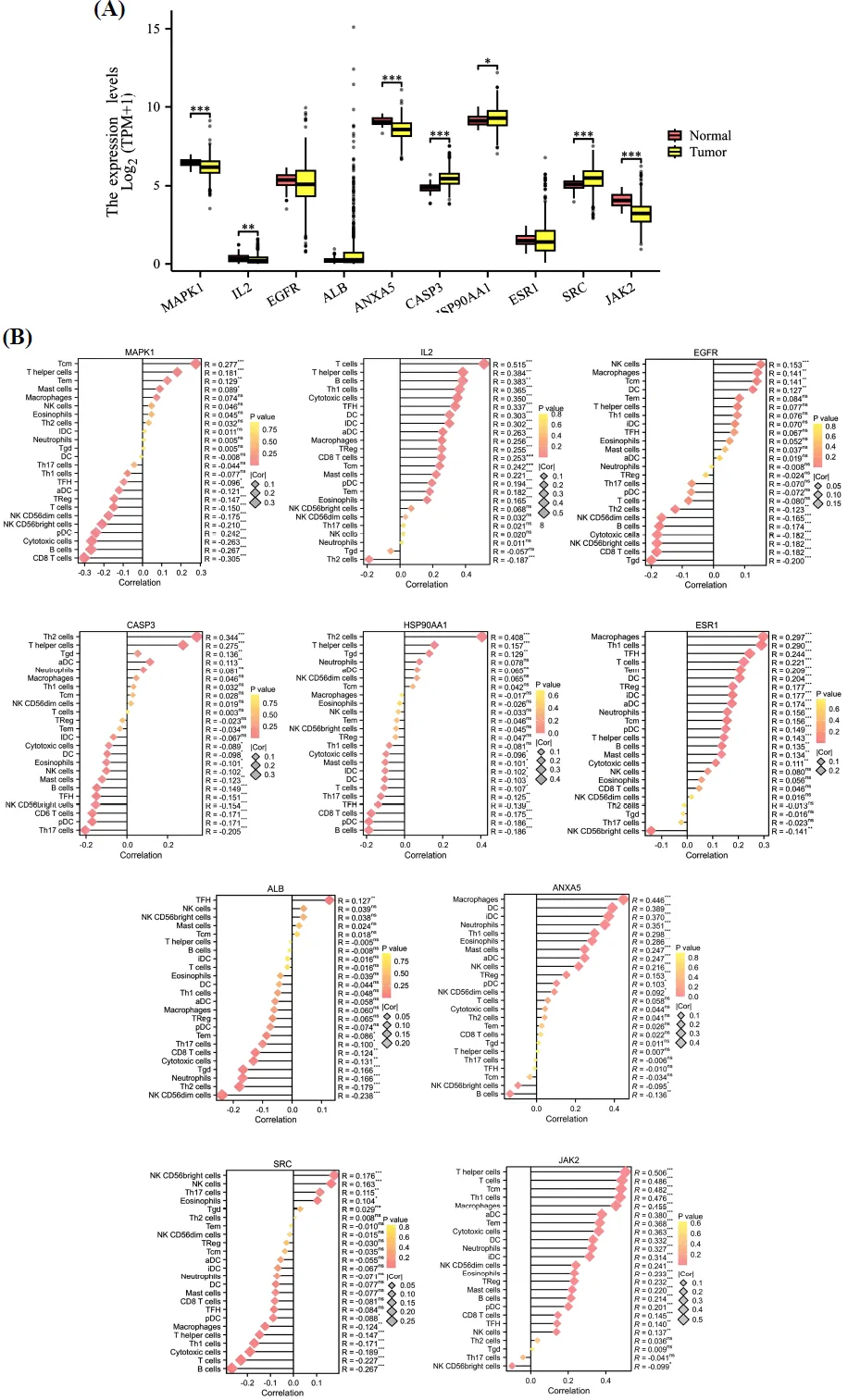
Expression of core targets for SAN treatment in LUAD (**A**) and their relationship with tumor immune cell infiltration in LUAD (**B**). aDC(activated DC); iDC(immature DC); pDC(Plasmacytoid DC);Tcm(T central memory); Tem(T effector memory); TFH(T follicular helper); Tgd(T gamma delta).

**Fig. (8):**
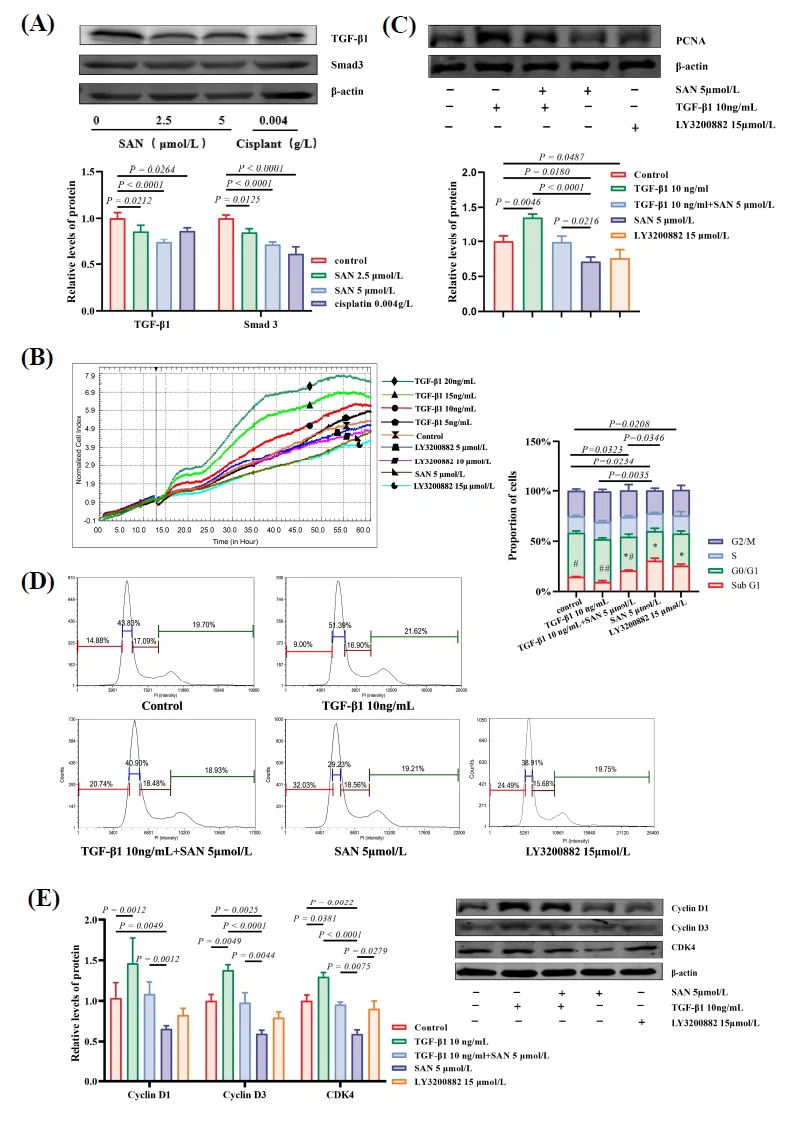
(**A**) SAN inhibited A549 cell Proliferation by inhibiting the TGF-β1/Smad3 pathway. Effect of SAN on TGF-β1 and Smad3 expression in A549 cells. (**B**) Impact of TGF-β1 on A549 cell proliferation. (**C**) Impact of TGF-β1 (10ng/ml) and SAN (5μmol/L) co-treatment on PCNA expression in A549 Cells. The 95% confidence interval for SAN *versus* TGF-β1+SAN is 0.03956 to 0.5191. (**D**) Effect of TGF-β1 (10ng/ml) and SAN (5μmol/L) co-treatment on A549 cell cycle distribution. The 95% confidence interval for SAN *versus* TGF-β1+SAN is -15.63 to -3.960. (**E**) Impact of TGF-β1 (10ng/ml) and SAN (5 μmol/L) co-treatment on Cyclin D1, Cyclin D3, and CDK4 expression in A549 cells. The 95% confidence interval for SAN *versus* TGF-β1+SAN is 0.1468 to 0.6465 (Cyclin D1), -0.0005286 to 0.49925 (Cyclin D3), -0.04053 to 0.4592 (CDK4). (Blank group: Control; TGF-β1: Activator group; TGF-β1 + SAN: Activator TGF-β1 and SAN combination group; SAN: Sanguinarine group; LY3200882: Inhibitor group; *Versus* the control group, **P<0.01，*P<0.05; *versus* the SAN group, *^##^P*<0.01，^#^*P*<0.05).

**Fig. (9):**
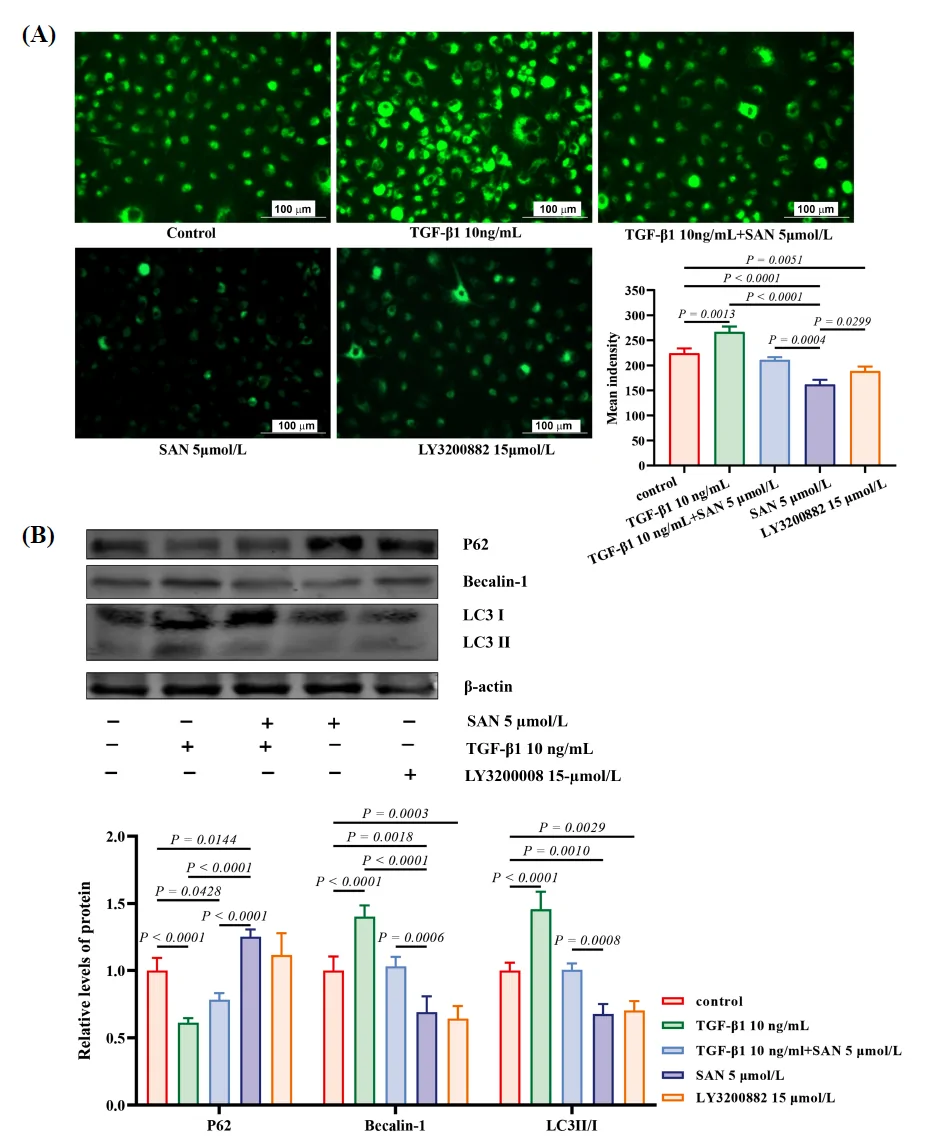
SAN inhibited A549 cell autophagy by inhibiting TGF-β1/Smad3 pathway. (**A**) Effects of TGF-β1 and SAN Co-treatment on A549 cell autophagy (MDC staining at 200x magnification). The 95% confidence interval for SAN *versus* TGF-β1+SAN is 33.89 to 72.11. (**B**) Impact of TGF-β1 and SAN Co-treatment on P62, Beclin-1, and LC3II/I in A549 cells. The 95% confidence interval for SAN *versus* TGF-β1+SAN is -0.6283 to -0.1891 (P62), 0.1207 to 0.5599 (Becalin-1), 0.1107 to 0.5499 (LC3II/I). (**A**) (Blank group: Control; TGF-β1: Activator group; TGF-β1 + SAN: Activator TGF-β1 and SAN combination group; SAN: Sanguinarine group; LY3200882: Inhibitor group; *Versus* the control group, **P<0.01, *P<0.05; *versus* the SAN group, ^##^*P*<0.01, ^#^*P*<0.05).

**Fig. (10):**
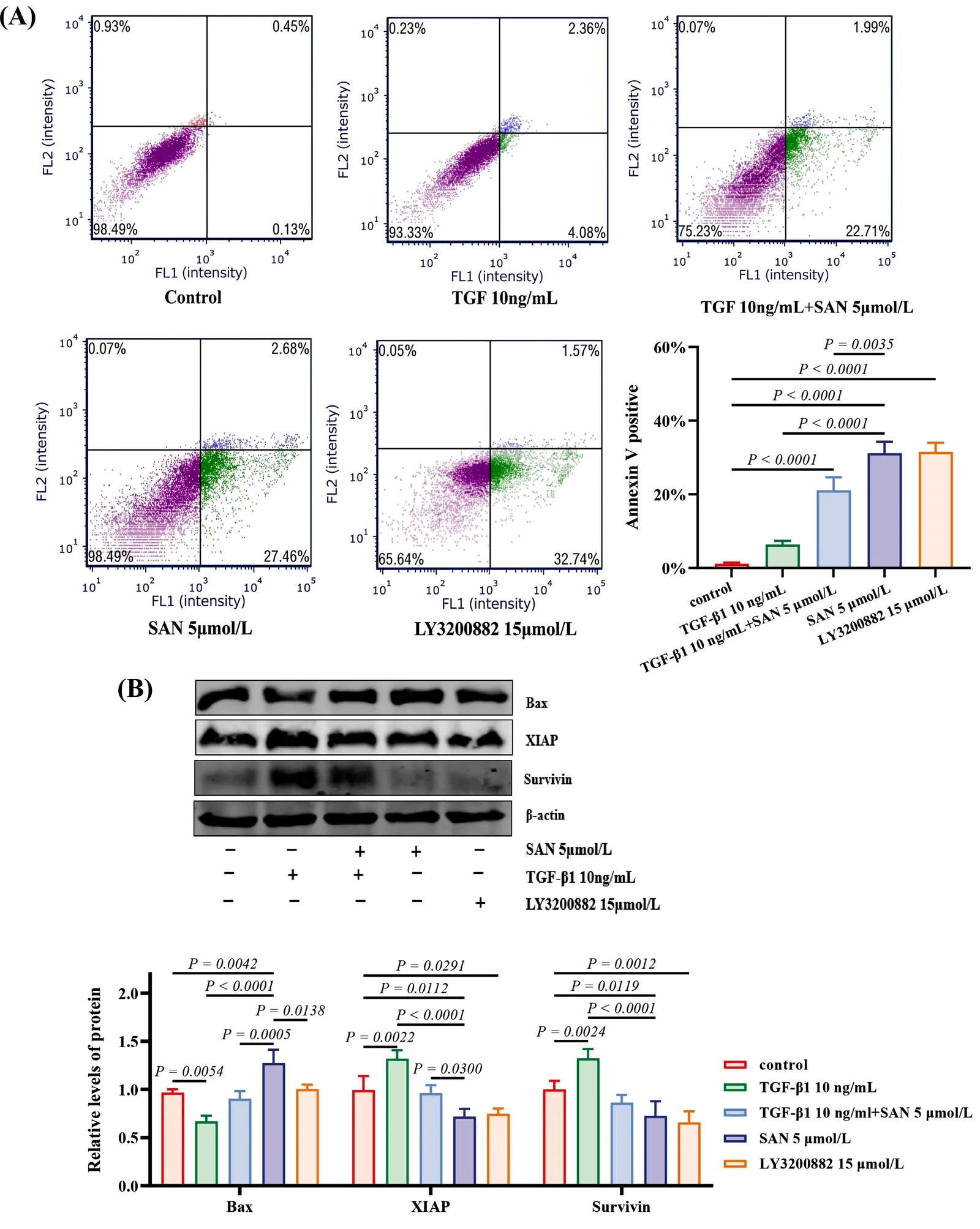
(**A**) SAN induced A549 cell apoptosis by inhibiting TGF-β1/Smad3 pathway. Effects of TGF-β1 and SAN co-treatment on A549 cell apoptosis. The 95% confidence interval for SAN *versus* TGF-β1+SAN is -16.64 to -3.526. (**B**) Impact of TGF-β1 and SAN co-treatment on Bax, XIAP, and Survivin expression in A549 cells. The 95% confidence interval for SAN *versus* TGF-β1+SAN is -0.5970 to -0.1423 (Bax), 0.01734 to 0.4720 (XIAP), and -0.09032 to 0.3643 (Survivin). (Blank group: Control; TGF-β1: Activator group; TGF-β1 + SAN: Activator TGF-β1 and SAN combination group; SAN: Sanguinarine group; LY3200882: Inhibitor group; *Versus* the control group, **P<0.01, *P<0.05; *versus* the SAN group, ^##^*P*<0.01, ^#^*P*<0.05).

**Table 1:** Online database names and URLs.

**Online Database**	**URLs**
DisGeNET	https://www.disgenet.org/
GeneCards	https://www.genecards.org/
OMIM	https://omim.org/
Venny 2.1	http://www.liuxiaoyuyuan.cn/
SwissTargetPrediction	http://www.swisstargetprediction.ch/
Pharm Mapper	http://www.lilab-ecust.cn/pharmmapper/

**Table 2:** Binding affinities of SAN with core targets as determined by docking.

**Core Target**	**PDB ID**	**Binding Energy (kCa/mol)**	**PyMOL**
MAPK1	8aoj	-8.5	MET108-2.2
IL2	8soz	-8.3	ARG81-2.7; ASN30-2.8
EGFR	8a27	-10.6	THR790-2.2
ALB	6yg9	-9.3	LYS432-2.3; LYS190-2.5
ANXA5	8h9z	-10.2	ARG271-2.4; LYS70-2.4
CASP3	2dko	-8.3	ARG149-2.8; LYS271-2.3
HSP90AA1	6tn5	-9.4	ASN45-2.5
ESR1	7baa	-8.8	SER45-2.6; SER45-2.3; LYS122-2.2
SRC	1fmk	-9.6	LYS152-2.1
JAK2	8bxh	-11.2	ARG938-2.5; ARG938-2.5

## Data Availability

All data generated or analyzed during this study are included in this published article.

## LIST OF ABBREVIATIONS

ANOVA: Analysis of Variance
BP: Biological Processes
CC: Cellular Components
FDA: Food and Drug Administration
FITC: Fluorescein Isothiocyanate
GO: Gene Ontology
KEGG: Kyoto Encyclopedia of Genes and Genomes
LUAD: Lung Adenocarcinoma
MDC: Monodansylcadaverine
MF: Molecular Functions
miRNA: MicroRNA
MSigDB: Molecular Signatures Database
NSCLC: Non-Small Cell Lung Cancer
PI: Propidium Iodide
PPI: Protein-Protein Interaction
RTCA: Real-Time Cellular Analysis
SAN: Sanguinarine
TCGA: The Cancer Genome Atlas

## References

[1] Bray F., Laversanne M., Sung H. (2024). Global cancer statistics 2022: GLOBOCAN estimates of incidence and mortality worldwide for 36 cancers in 185 countries.. CA Cancer J Clin.

[2] Arbour K.C., Riely G.J. (2019). Systemic therapy for locally advanced and metastatic non–small cell lung cancer.. JAMA.

[3] Zielińska S., Dziągwa-Becker M., Junka A. (2021). Screening papaveraceae as novel antibiofilm natural-based agents.. Molecules.

[4] Ali I., Li J., Cui L., Zhao H., He Q., Wang D. (2021). Efficient extraction and purification of benzo[ c ]phenanthridine alkaloids from Macleaya cordata (Willd) R. Br. by combination of ultrahigh pressure extraction and pH‐zone‐refining counter‐current chromatography with anti‐breast cancer activity in vitro.. Phytochem Anal.

[5] Sun W., Xu Y., Liu Z. (2025). Studies on pharmacokinetic properties and intestinal absorption mechanism of sanguinarine chloride: In vivo and in situ.. Toxicol Mech Methods.

[6] Zhong L.T., Yuan J.M., Fu W.L. (2024). Identification of sanguinarine as c-MYC transcription inhibitor through enhancing the G-quadruplex-NM23-H2 interactions.. Bioorg Chem.

[7] Ullah A., Razzaq A., Alfaifi M.Y. (2024). Sanguinarine attenuates lung cancer progression via oxidative stress-induced cell apoptosis.. Curr Mol Pharmacol.

[8] Messeha S.S., Noel S., Zarmouh N.O., Womble T., Latinwo L.M., Soliman K.F.A. (2023). Involvement of AKT/PI3K pathway in sanguinarine’s induced apoptosis and cell cycle arrest in triple-negative breast cancer cells.. Cancer Genomics Proteomics.

[9] Hu X., Li G., Kong C. (2025). Fluorinated chitosan mediated transepithelial delivery of sanguinarine-loaded platinum (IV) prodrug for intravesical instillation therapy of muscle-invasive bladder cancer.. J Control Release.

[10] Ding Q., Zhu W., Zhu S., Zhou X. (2022). Sanguinarine promotes apoptosis of hepatocellular carcinoma cells via regulating the miR-497-5p/CDK4 axis.. Am J Transl Res.

[11] Qin T.T., Li Z.H., Li L.X. (2022). Sanguinarine, identified as a natural alkaloid LSD1 inhibitor, suppresses lung cancer cell growth and migration.. Iran J Basic Med Sci.

[12] Wei X., Hou W., Liang J. (2021). Network pharmacology-based analysis on the potential biological mechanisms of sinisan against non-alcoholic fatty liver disease.. Front Pharmacol.

[13] Daina A., Michielin O., Zoete V. (2019). SwissTargetPrediction: updated data and new features for efficient prediction of protein targets of small molecules.. Nucleic Acids Res.

[14] Wang X., Shen Y., Wang S. (2017). PharmMapper 2017 update: a web server for potential drug target identification with a comprehensive target pharmacophore database.. Nucleic Acids Res.

[15] Stelzer G., Rosen N., Plaschkes I. (2016). The GeneCards suite: From gene data mining to disease genome sequence analyses.. Curr Protoc Bioinformatics.

[16] Amberger J.S., Bocchini C.A., Schiettecatte F., Scott A.F., Hamosh A. (2015). OMIM.org: Online mendelian inheritance in man (OMIM®), an online catalog of human genes and genetic disorders.. Nucleic Acids Res.

[17] Piñero J., Bravo À., Queralt-Rosinach N. (2017). DisGeNET: A comprehensive platform integrating information on human disease-associated genes and variants.. Nucleic Acids Res.

[18] Mering C., Huynen M., Jaeggi D., Schmidt S., Bork P., Snel B. (2003). STRING: A database of predicted functional associations between proteins.. Nucleic Acids Res.

[19] Shannon P., Markiel A., Ozier O. (2003). Cytoscape: A software environment for integrated models of biomolecular interaction networks.. Genome Res.

[20] Dennis G., Sherman B.T., Hosack D.A. (2003). DAVID: Database for annotation, visualization, and integrated discovery.. Genome Biol.

[21] (2006). The gene ontology (GO) project in 2006.. Nucleic Acids Res.

[22] Chen E.Y., Tan C.M., Kou Y. (2013). Enrichr: Interactive and collaborative HTML5 gene list enrichment analysis tool.. BMC Bioinformatics.

[23] Kuleshov M.V., Jones M.R., Rouillard A.D. (2016). Enrichr: A comprehensive gene set enrichment analysis web server 2016 update.. Nucleic Acids Res.

[24] Xie Z., Bailey A., Kuleshov M.V. (2021). Gene set knowledge discovery with enrichr.. Curr Protoc.

[25] Trott O., Olson A.J. (2010). AutoDock vina: Improving the speed and accuracy of docking with a new scoring function, efficient optimization, and multithreading.. J Comput Chem.

[26] Hänzelmann S., Castelo R., Guinney J. (2013). GSVA: gene set variation analysis for microarray and RNA-Seq data.. BMC Bioinformatics.

[27] Bindea G., Mlecnik B., Tosolini M. (2013). Spatiotemporal dynamics of intratumoral immune cells reveal the immune landscape in human cancer.. Immunity.

[28] Zhang G., Zhu L., Xue Y. (2020). Benzophenanthridine alkaloids suppress lung adenocarcinoma by blocking TMEM16A Ca2+-activated Cl− channels.. Pflugers Arch.

[29] Saini N., Grewal A.S., Lather V., Gahlawat S.K. (2022). Natural alkaloids targeting EGFR in non-small cell lung cancer: Molecular docking and ADMET predictions.. Chem Biol Interact.

[30] Yu Y., Luo Y., Fang Z. (2020). Mechanism of sanguinarine in inhibiting macrophages to promote metastasis and proliferation of lung cancer via modulating the exosomes in A549 Cells.. OncoTargets Ther.

[31] Prabhu K.S., Bhat A.A., Siveen K.S. (2021). Sanguinarine mediated apoptosis in Non-Small Cell Lung Cancer via generation of reactive oxygen species and suppression of JAK/STAT pathway.. Biomed Pharmacother.

[32] Cui Y., Luo Y., Qian Q. (2022). Sanguinarine regulates tumor-associated macrophages to prevent lung cancer angiogenesis through the WNT/β-catenin pathway.. Front Oncol.

[33] Zhang S., Leng T., Zhang Q., Zhao Q., Nie X., Yang L. (2018). Sanguinarine inhibits epithelial ovarian cancer development via regulating long non-coding RNA CASC2-EIF4A3 axis and/or inhibiting NF-κB signaling or PI3K/AKT/mTOR pathway.. Biomed Pharmacother.

[34] Lee T.K., Park C., Jeong S.J. (2016). Sanguinarine induces apoptosis of human oral squamous cell carcinoma KB Cells via inactivation of the PI3K/Akt signaling pathway.. Drug Dev Res.

[35] Qi X., Chen Y., Liu S. (2023). Sanguinarine inhibits melanoma invasion and migration by targeting the FAK/PI3K/AKT/mTOR signalling pathway.. Pharm Biol.

[36] Xu X., Deng L., Tang Y. (2023). Cytostatic activity of sanguinarine and a cyanide derivative in human erythroleukemia cells is mediated by suppression of c-MET/MAPK Signaling.. Int J Mol Sci.

[37] Lee B., Lee S.J., Park S.S. (2008). Sanguinarine-induced G1-phase arrest of the cell cycle results from increased p27KIP1 expression mediated via activation of the Ras/ERK signaling pathway in vascular smooth muscle cells.. Arch Biochem Biophys.

[38] Garg P., Pareek S., Kulkarni P., Horne D., Salgia R., Singhal S.S. (2025). Exploring the potential of TGFβ as a diagnostic marker and therapeutic target against cancer.. Biochem Pharmacol.

[39] Peng R., Li Y., Gao Y., Chen D., Li Z., Zhao Y. (2024). Cellular and molecular landscape of primary dermatofibrosarcoma protuberans: Insights from single‐cell RNA sequencing analysis.. Exp Dermatol.

[40] Lan X., Wei D., Fang L., Wu X., Wu B. (2024). Tumor-associated macrophage-derived TGF-β1 activates GLI2 via the Smad2/3 signaling pathway to affect cisplatin resistance in lung adenocarcinoma.. Technol Cancer Res Treat.

