# Supplementary material for: Sanguinarine Suppresses Lung Adenocarcinoma via TGF-β1/Smad3 Path way: Insights from Network Analysis and Experimental Validation
Source: Curr Pharm Des. 2026 Jan 9;32(35):2953–68. doi: 10.2174/0113816128404840251114063502 (PMC13698023; doi:10.2174/0113816128404840251114063502)
Supplement: Supplementary file 1 [file CPD-32-35-2953_SD1.zip › CPD-32-35-2953_SD1/CPD-32-35-04-Supply.pdf]

**Supplementary Material**

# Sanguinarine Suppresses Lung Adenocarcinoma *via* TGF-β1/Smad3 Pathway: Insights from Network Analysis and Experimental Validation

Rong Yuan[1,2,#], Xiaona Li[1,3,#], Junqi Wang[1,2], Zhaojun Xu[2,4,*] and Lan Song[1,2,*]

*[1]Medicine School, Hunan University of Chinese Medicine, 300 Xueshi Road, Hanpu Science and Teaching Park, Changsha, Hunan, 410208, China; [2]Hunan Provincial Key Laboratory of Vascular Biology and Translational Medicine, 300 Xueshi Road, Hanpu Science and Teaching Park, Changsha, Hunan, 410208, China; [3]Hebei Yuzhilin Biotechnology Co., Ltd., No. 168 Haidian Road, Shijiazhuang, Hebei, 050035, China; [4]Cardiothoracic Surgery of the First Affiliated Hospital, Hunan University of Chinese Medicine, 97 Shaoshan Road, Changsha, Hunan, 410007, China*

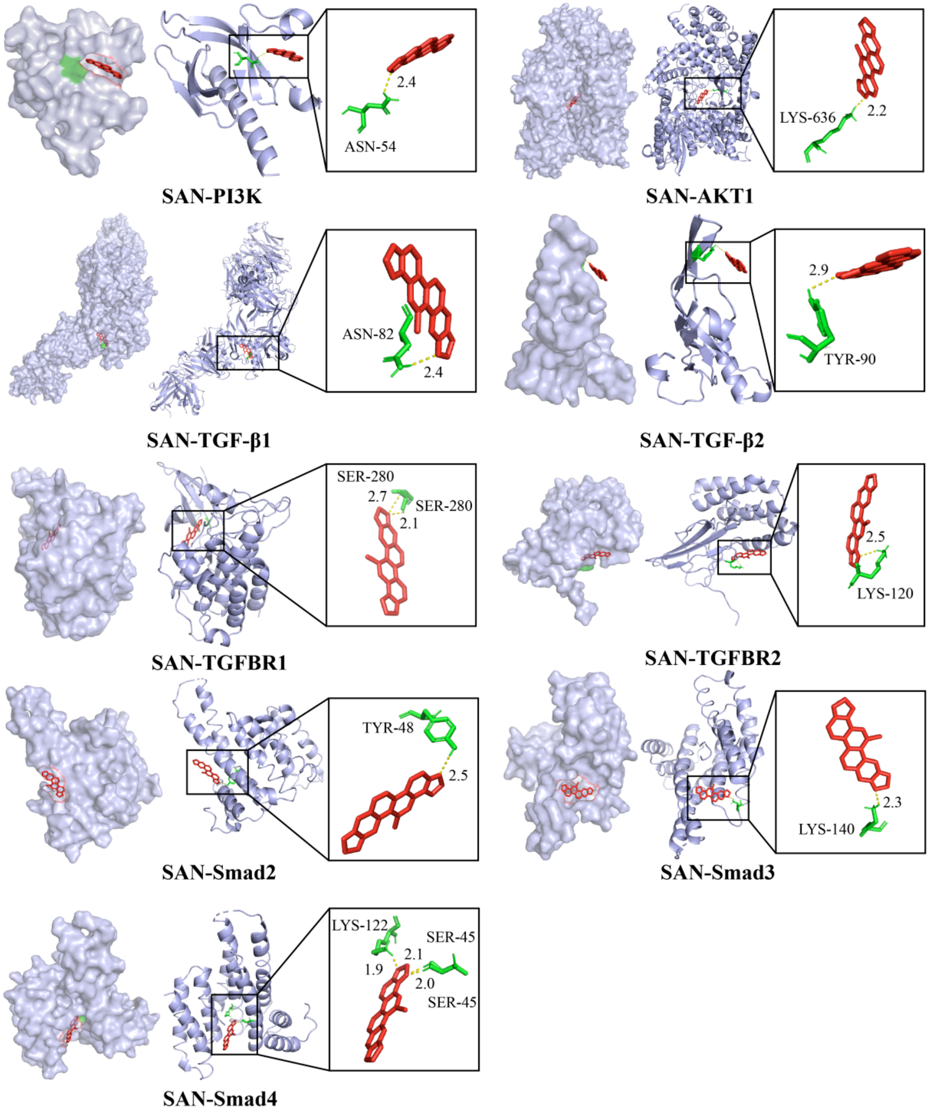


**Fig. (S1). Molecular Docking Visualization of SAN with PI3K/AKT and TGF-β pathway**

**1873-4286/26 $65.00+.00** **© 2026 Bentham Science Publishers**

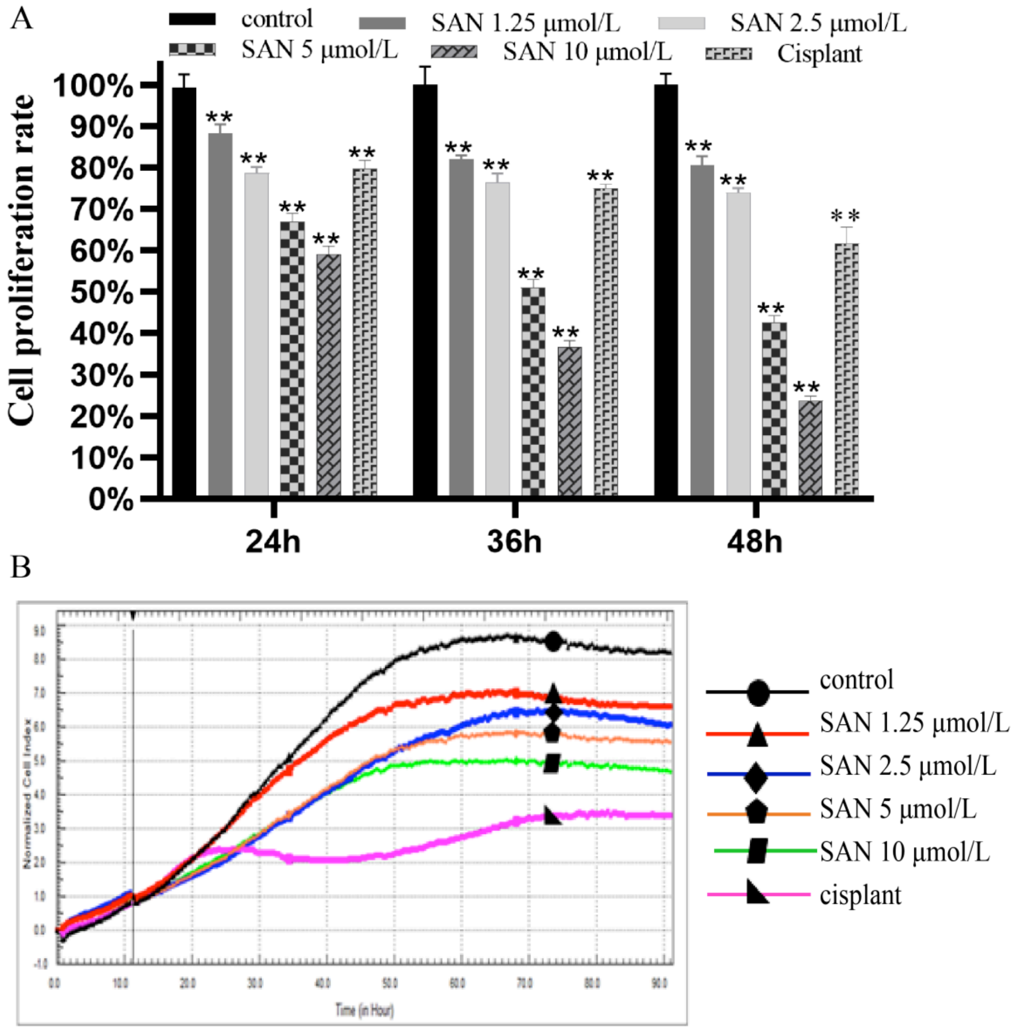


**Fig. (S2). SAN inhibits the proliferation of A549 cells. (A). Effect of SAN on A549 cell proliferation was detected by MTT assay. (B). Effect of SAN on proliferation of A549 cells was monitored by RTCA. (versus the control, *****P*<0.01, n=3)**

Combined with the results of MTT and RTCA experiments, low concentration SAN ( 2.5, 5 μmol/L ) with better inhibition of proliferation was selected to treat A549 cells for subsequent experiments.

Note:
Fig. (S2 B) was published in "*Li Xiaona, et al. Preliminary study on the mechanism of sanguinarine inhibiting the growth of lung adenocarcinoma A549 cells. Journal of Hunan University of Traditional Chinese Medicine 41.01 ( 2021 ) : 61-66.*".
Literature link :
https://kns.cnki.net/kcms2/article/abstract?v=oWJgMrFo8ufzuki6bSJG71KqqmaxM6tBLI5ChFfReSWaSuTUzWS4GL6pY6A4ykxtc7UZt_m9mXLph1NXMVI4pufTZJDhvaSAEa5Dtb78DFkjfdqBwrWRsP_naU6QhPMfbJYPLB09Y7njNUdaGi1MxKAFtRFgTvtJbbTjsWGRzCUszcxSg-X565aw_GcBfeDLBx3MADCMkiI=&uniplatform=NZKPT&language=CHS

**Table S4. Binding Affinities of SAN with PI3K/AKT and TGF-β pathway as Determined by Docking.**

| Core target | PDB ID | Binding Energy (kCa/mol) | PyMOL |
|---|---|---|---|
| AKT1 | 1unq | -7.1 | ASN54-2.4 |
| PI3K | 7rsp | -8.5 | LYS636-2.2 |
| TGF-β1 | 8udz | -8.8 | ASN82-2.4 |
| TGF-β2 | 2tgi | -6.8 | TYR-90 |
| TGFBR1 | 5e8s | -10.4 | SER280-2.1, 2.7 |
| TGFBR2 | 8g4k | -8.3 | LYS120-2.5 |
| Smad2 | 6yia | -8.6 | TYR48-2.5 |
| Smad3 | 6yib | -8.9 | LYS140-2.3 |
| Smad4 | 6yic | -8.6 | SER45-2.1, 2.0; LYS122-1.9 |
